# Humpback whale song revolutions continue to spread from the central into the eastern South Pacific

**DOI:** 10.1098/rsos.220158

**Published:** 2022-08-31

**Authors:** Josephine N. Schulze, Judith Denkinger, Javier Oña, M. Michael Poole, Ellen C. Garland

**Affiliations:** ^1^ Sea Mammal Research Unit (SMRU), Scottish Oceans Institute, School of Biology, University of St Andrews, St Andrews, Fife KY16 8LB, UK; ^2^ Colegio de Ciencias Biológicas y Ambientales (Cociba), Universidad San Francisco de Quito, Quito EC170157, Ecuador; ^3^ Acoustic Ecology Program, CETACEA Ecuador Project, Quito EC17015, Ecuador; ^4^ Marine Mammal Research Program, BP 698, Maharepa, 98728 Moorea, French Polynesia; ^5^ Centre for Social Learning and Cognitive Evolution, School of Biology, University of St Andrews, St Andrews, Fife KY16 9TH, UK

**Keywords:** cultural transmission, song, vocal learning, cultural evolution, humpback whale, South Pacific

## Abstract

Cultural transmission of behaviour is an important aspect of many animal communities ranging from humans to birds. Male humpback whales (*Megaptera novaeangliae*) sing a repetitive, stereotyped, socially learnt and culturally transmitted song display that slowly evolves each year. Most males within a population sing the same, slow-evolving song type; but in the South Pacific, song ‘revolutions’ have led to rapid and complete replacement of one song type by another introduced from a neighbouring population. Songs spread eastwards, from eastern Australia to French Polynesia, but the easterly extent of this transmission was unknown. Here, we investigated whether song revolutions continue to spread from the central (French Polynesia) into the eastern (Ecuador) South Pacific region. Similarity analyses using three consecutive years of song data (2016–2018) revealed that song themes recorded in 2016–2018 French Polynesian song matched song themes sung in 2018 Ecuadorian song, suggesting continued easterly transmission of song to Ecuador, and vocal connectivity across the entire South Pacific Ocean basin. This study demonstrates songs first identified in western populations can be transmitted across the entire South Pacific, supporting the potential for a circumpolar Southern Hemisphere cultural transmission of song and a vocal culture rivalled in its extent only by our own.

## 1. Introduction

Cultural traditions are significant to human society [1], but also shape non-human mammalian societies including primates, rats and cetaceans, and non-mammalian species such as fish and birds [2–10]. Vocally learnt displays play an important role in shaping culture in oscine birds and cetaceans, in particular [11–14]. Culture is defined here, following others, as the social learning of information or behaviours from conspecifics within a community [10,15–17]. Information can flow in a number of different directions. Horizontal transmission is within-generation spread of cultural traditions, while vertical transmission occurs from parent to offspring, and oblique transmission is spread from non-parental individuals belonging to the previous generation to the next generation [10,18,19].

Song is a striking example of non-human cultural transmission and evolution exhibited by oscine songbirds and possibly most baleen whales including humpback whales [12,20]. Bird song is a crucial part of courtship behaviour, and despite some basic song structure being innate, the complexity and detail are added through contact with conspecifics [21]. Further, some bird songs undergo changes from year to year by individuals dropping and adding syllables [22,23]. Corn buntings (*Emberiza (Miliaria) calandra*), for example, possess local dialects that are distinct from conspecifics beyond a geographical boundary [24,25]. The song of a certain dialect changes slowly each year, with all males adopting the novel version in unison [12,24,26]. This process of small changes through individuals performing their own rendition of the song is described as cultural evolution [27]. Song is also thought to play a role in breeding success of baleen whales [28–30]. The song produced by baleen whales ranges in complexity in terms of the number of sound types, structure and length. Blue whales (*Balaenoptera musculus*) and fin whales (*Balaenoptera physalus*) sing simple songs made up of only a few sound types [31,32]. By contrast, bowhead whales (*Balaena mysticetus*) and humpback whales sing complex songs that change over breeding seasons [30,33–35]. Unlike bowhead whale song where many song types appear to be present in one season [34,36], humpback whales within a population typically sing a single, shared song type that progressively evolves each year, much like that of corn buntings, but there are notable exceptions (see below) [29,30,37,38].

**Figure 1.**
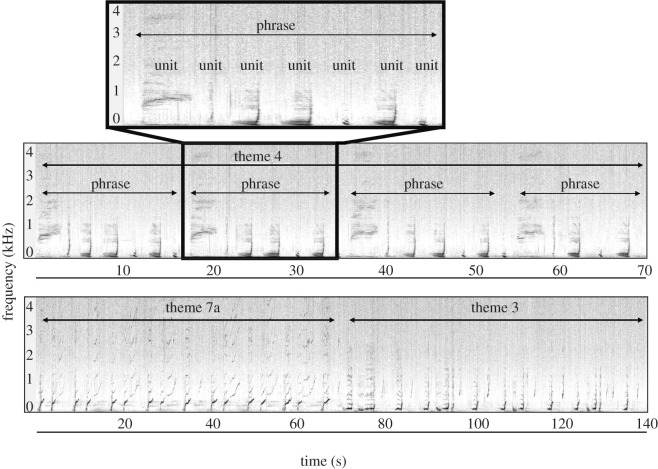
Humpback whale song hierarchy depicted using spectrograms of themes 4, 7a and 3 from 2016 French Polynesia (singer 2). Several ‘units’ make up a ‘phrase’, which when repeated comprise a ‘theme’, and several themes create a ‘song’ [30]. Spectrograms were made in Raven Pro 1.6.1 (fast Fourier transform 2048, Hann window, 50% overlap).

Humpback whale song is arranged in a nested hierarchy with multiple levels [30,39]. At the lowest level, each vocalization (defined as the shortest continuous sound to our ears) is called a ‘unit’ (figure 1). A few units are combined to form a string of units called a ‘phrase’. Multiple repeating phrases create a ‘theme’, and several themes sung in a specific order form a ‘song’ that typically lasts between five and 30 min [30,40,41]. Repeating songs are called ‘song sessions’ that can extend over several hours [30]. At the highest level, songs are classified into ‘song types’ based on their theme structure and order [18]. Unlike social sounds that are produced by both females and males [42,43], only adult male humpback whales engage in singing [30,39,44]. This highly stereotyped, repetitive and progressively evolving song occurs primarily on winter breeding grounds and during migration [39,44–46]. Humpback whales spend their summers in high latitude feeding grounds, where they forage [47–49]. After summer, humpbacks undertake long migrations to low latitude winter breeding grounds to rest, mate and give birth [39,48,50]. The function of song remains contested, although it is thought to play an important role in male breeding success [51]. Whether it serves as a courtship behaviour targeted toward females [46], to mediate male–male interaction [51–53], or as a multi-message signal, remains unclear [51,54].

In the South Pacific region, changes to humpback whale song may occur in two ways: progressive cultural evolution, and cultural ‘revolution’ [41]. Progressive evolution may occur when males embellish their own song through addition, substitution or deletion at any hierarchical level and through copying changes from other males [12,44,55]. Since song is learnt from conspecifics, this individual variation results in the same song slowly evolving over several breeding seasons [39]. By contrast, cultural ‘revolutions’ describe rapid replacement of one song type with an entirely novel one introduced from a neighbouring population, although the underlying mechanism(s) driving this phenomenon remain elusive [41,56]. Noad *et al*. [56] first observed a cultural revolution of humpback whale song spreading from one population to another in Australia. The eastern Australian population of whales adopted the song previously sung in western Australia in a time span of merely 2 years—a time frame far too brief to be considered progressive evolution [56]. Subsequent work has shown that humpback whale song revolutions continue to spread in a unidirectional pattern eastward from eastern Australia across to French Polynesia [41,57,58]. One song type described in eastern Australia in 2003 was tracked across multiple genetically distinct breeding populations and arrived in French Polynesia in 2005, with this pattern of transmission consistent for several different song types [41,56,59]. In some rare occasions, hybrid songs containing themes from both old and new song types were recorded during seasons of song revolutions [60]. Such instances of hybrid songs were suggested to be active song learning by a whale that is switching from singing the old song type to the new one [60]. Revolutionary songs appear simpler (less complex in terms of number of unit types, length and number of themes) than the songs they replace [61]. The underlying mechanism of transmission remains unclear, particularly given the genetic distinction between populations and strong site fidelity [59,62,63]. However, the following mechanisms may allow song transmission [55]: individuals moving from one breeding population to another (1) within or (2) between seasons may introduce new songs, or (3) song may be exchanged on shared or partially shared migration routes as well as summer feeding grounds (e.g. around Antarctica) [55,57,60,64]. Recently, a location in the western South Pacific—the Kermadec Islands—was suggested to have served as a stopover for humpback whales from multiple populations during their southward migration [58]. Similarities in song themes linked the Kermadec Islands to several wintering grounds, and provided the first indications of where cultural transmission between otherwise acoustically isolated populations on the winter breeding grounds may take place [58] (as song transmits for tens of kilometres [65,66]).

**Figure 2.**
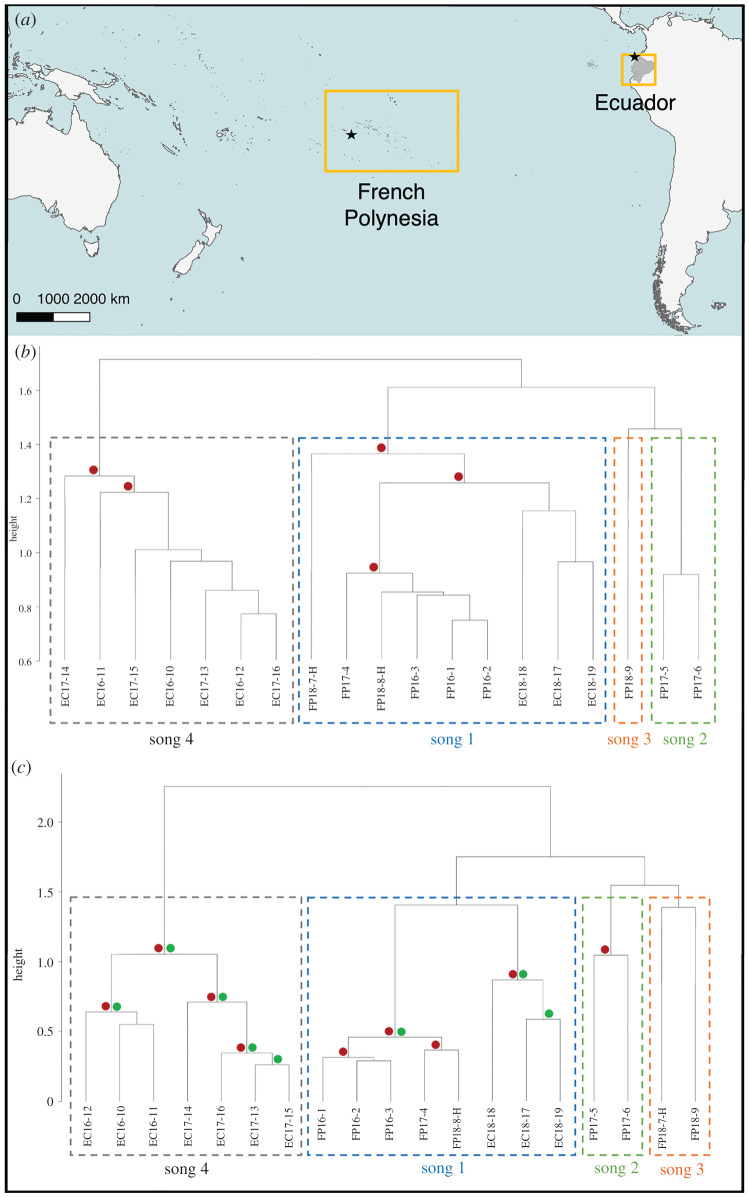
(*a*) Map of South Pacific showing study locations: French Polynesia (Mo'orea; star) and Ecuador (Esmeraldas; star), spanning ∼8000 km between them. (*b*) Dendrogram of bootstrapped (1000) average-linkage hierarchically clustered songs among singers in French Polynesia (FP) and Ecuador (EC) each year (2016, 2017, 2018) for LSI using theme sequences (CCC = 0.962), and (*c*) DSI using theme presence (CCC = 0.966). Hybrid singers (singers 7 and 8) labelled with H (table 1). Singer 7 combined themes from song types 1 (blue) and 3 (orange). Singer 8 combined themes from song types 1 (blue) and 2 (green). Singer label is constructed as the location, year, and singer number. Red dots indicate AU (multiscale resampling) *p*-values greater than 95% where divisions were stable and likely to occur. Green dots indicate BP (normal bootstrap probability) *p*-values greater than 70% suggesting that tree structure and branching was stable and likely to occur. Dashed boxes indicate the clustering of each song type.

The vocal connectivity of western and central South Pacific populations, and the consistent, unidirectional spread of humpback whale songs from western Australia to French Polynesia raise the question of how far a song may be transmitted eastwards [41,58,67,68]. The distance from western Australia to French Polynesia spans just under 10 000 km, with multiple ‘populations’ of whales (specifically, eastern Australia, New Caledonia, Tonga, American Samoa and the Cook Islands) located in between [41,69]. However, the next major population of humpbacks eastward of French Polynesia migrates along the west coast of South America to breeding grounds located primarily off Ecuador and Colombia, about 8000 km away [69] (figure 2*a*). Here, we investigated whether song revolutions continue to spread in a unidirectional pattern eastward from the central South Pacific (French Polynesia) into the eastern South Pacific (Ecuador) region. Using similarity analyses (Levenshtein distance similarity index (LSI) and Dice's similarity index (DSI)) songs from three consecutive and concurrent years (2016–2018) were quantitatively compared to investigate the direction and strength of connection. The geographical bounds of song transmission are unknown but are hypothesized to be circumpolar, based on recent cultural evolution models [70]. This current paper employing empirical data provides the first understanding of connectivity across the entire South Pacific Ocean basin.

## 2. Material and methods

### 2.1. Song recordings

Song recordings were collected on the humpback whale breeding grounds in French Polynesia off the island of Mo'orea, and in northern Ecuador off the coast of Esmeraldas (figure 2*a*; electronic supplementary material, figure S1). Recordings were made during the austral winter breeding season (July–November) from 2016 to 2018 during opportunistic boat-based surveys and with a moored autonomous recorder. Boat-based recordings were made in French Polynesia in 2016 using a HTI 96MIN hydrophone connected to a H4N Pro Zoom recorder (WAV format, 16 bit, sampling rate 44.1 kHz). Passive acoustic recordings were collected in 2016, 2017 and 2018 using an Ocean Instruments SoundTrap STD300 (WAV format, 16 bit, sampling rate 24 kHz, duty cycle 30 min in every 120 min) in 30 m water (recorder 4 m above the sea floor) at 17°32.860 S and 149°46.148 W (electronic supplementary material, figure S1b). All recordings in Ecuador were boat-based and made in the ‘Bajos de Atacames’ up to 5–10 km offshore from the Esmeraldas River (0°59054.1″ N, 79°38037.7″ W) to Punta Galera (0°49010.15″ N, 80°02055.67″ W; electronic supplementary material, figure S1c). Songs were recorded using a H2a-XLR omnidirectional hydrophone (sensitivity of –180 dBV/uPa +4 dB, from 20 Hz to 100 kHz) and a DolphinEar/Pro omnidirectional hydrophone (sensitivity 15 Hz to 20.000 Hz ± 3 dB) connected to a TASCAM DR-40 recorder (WAV format, 16 bit, sampling rate 44.1 kHz).

The data represent a snapshot of song sung in each of these populations each year but are broadly representative of each population due to the strong song matching among individuals [41,58]. The three highest quality recordings were selected from each year (representing the start, middle and end of the season, where possible) in French Polynesia and Ecuador (table 1, *N* = 18; see electronic supplementary material, S1). One recording (from Ecuador 2017, table 1) had multiple singers present in the recording; it was uncertain that the same individual remained in the foreground consistently, so the recording was subdivided into sections where the song could consistently be followed. This resulted in four sections of song which were treated as ‘individual singers’, but two of these were not included in song comparisons due to being less than 10 min in length and thus may not be representative of a full song. As a result, a total of 21 singers from 18 recordings were transcribed, with 19 singers in total being included in song comparison analyses (table 1).

**Table 1.** Song recordings from two South Pacific breeding populations, French Polynesia and Ecuador. A total of 18 song recordings were analysed between 2016 and 2018. The number of song cycles (no repetition of a theme but allowing repetition of phrase variants/types if consecutive), the song type (song type 1 = blue, song type 2 = green, song type 3 = orange, song type 4 = grey) and the sequence of themes sung (*theme descriptions are in electronic supplementary material, table S2) are noted per singer. H = hybrid singer combining themes from two song types [60].

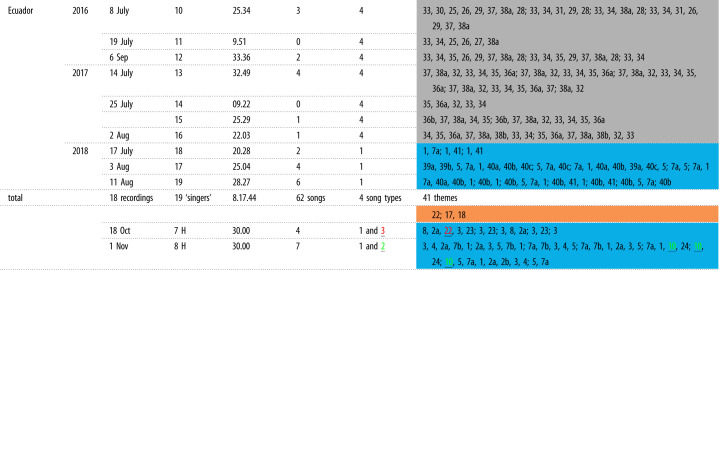

### 2.2. Song transcription and unit classification

All songs were analysed as spectrograms in Raven Pro 1.6.1 (fast Fourier transform 2048, Hann window, 50% overlap, showing 0–5 kHz and 20 s increments). Song transcription was conducted at the unit level by a human classifier (J.N.S.) following Garland *et al.* [40,41,57,71]. Each unit was classified based on its aural and visual characteristics such as frequency range, duration and contour. To ensure unit classifications were consistent and repeatable, a subset of units were measured for 11 acoustic parameters following previous humpback unit classification analyses [42,71,72] (see electronic supplementary material, S1, for further information). The subset of units selected for measurement (*n* = 859) were all units from one high-quality example of each phrase type present for each location and year, plus any rare unit type not included in those selected phrases [72]. A random forest analysis was run in R (v. 3.5.3) [73] using the *randomForest* package [74] (mtry = 6, 1000 trees) which resulted in an out-of-bag error rate of 27.47% indicating an adequate level of agreement between quantitative and qualitative classification of unit types.

### 2.3. Assigning unit strings to themes

Units were transcribed into phrases. Repeating unit strings (phrases) were grouped into themes following the typical theme classification rule of ‘similar sounds in similar positions' [37,66], and given a number (e.g. 1, 2, 3). Variations of the same theme which were comprised of the same or similar structure, but with slightly different units (e.g. ‘squeaks’ versus ‘high squeaks’; electronic supplementary material, table S2), or added/deleted units, were allocated letters (e.g. a, b, c) to indicate this variation (referred to as a ‘phrase type’, e.g. 7a). To ensure the qualitative assignment of phrases to themes was robust and repeatable for all themes regardless of the location or year they were recorded, a LSI analysis was undertaken, following previous studies [40,57,58,71,72]. The Levenshtein distance is a metric that quantifies the similarity between sequences through grouping or clustering those that are the most similar and has frequently been used to analyse the similarity of humpback whale song [51,72,74–77]. LSI produces a proportion of similarity between any two strings of data by counting the number of changes (additions, deletions or substitutions) to convert one string into another which is standardized for string length [40,66,71,78,79]. The LSI analysis was conducted in R using custom-written code (package *leven*, available at http://github.com/ellengarland/leven) to compare all phrase strings (*n* = 1457). The LSI theme similarity matrix was hierarchically clustered and visualized as a dendrogram using average-linkage (UPGMA) clustering to validate the qualitative assignment of phrases to themes (both within and between all years and populations). The cophenetic correlation coefficient (CCC) was also calculated to ensure the clustering method chosen provided the best representation of the connections within the data (considered ‘good’ if CCC > 0.8) [71,72,80]. The CCC using average-linkage clustering was 0.88 indicating our theme assignments were robust and were a good representation of the connections within the data. Once theme assignments were confirmed, a single set median unit string was calculated per theme for each location/year combination [40,78]. The calculation sums all similarity scores within the theme and selects the string with the highest score (i.e. similarity) to act as the most representative unit string for each theme, per location and year (electronic supplementary material, table S1).

### 2.4. Matching songs within and between populations

To investigate song sharing, we calculated two similarity indices: (1) the LSI and (2) DSI (see the following paragraph). Once theme assignments were verified, the LSI calculation was performed at the song level as the sequence of themes that made up each song type (table 1). For the LSI, phrase repetitions were omitted to extract the order of all themes per singer, regardless of the length of song. We made no attempt to divide the sequence of themes into individual songs for the analysis. The LSI was calculated for all theme sequences (*n* = 19) and the resulting similarity matrix was average-linkage clustered (and CCC calculated as above) and bootstrapped 1000 times (with multiscale bootstrap resampling (AU) and normal bootstrap probability (BP)) using the *pvclust* package [81] in R. Bootstrapping produced *p*-values for each split in the tree, which are regarded as significant if *p* > 95% for AU [81] and considered significant if *p* > 70% for BP [40] to ensure the tree was robust and stable.

As an alternative analysis to LSI, DSI was calculated based on the presence and sharing of themes (phrase types) among all singers. This analysis does not rely on any sequence information in the song, simply the presence and sharing of themes [82]. DSI is calculated as the number of shared themes divided by the sum of the total number of themes present in the song of singer 1 and singer 2 [82] (see electronic supplementary information, S1). DSI calculation was performed in R using custom-written code (available at https://github.com/ellengarland/dice_si). The similarity matrix was clustered (average-linkage) and bootstrapped 1000 times (as for the LSI), and the CCC calculated to ensure the resultant tree structure represented the connections in the data.

## 3. Results

Four song types (labelled 1–4) identified from 19 singers from French Polynesia (*n* = 9) and Ecuador (*n* = 10) were composed of 41 themes (table 1; electronic supplementary material, tables S1 and S3). Song type 1 (coloured blue; electronic supplementary material, figures S2–S4 and S7) was identified in French Polynesia in 2016 (3/3 singers) and was also present in French Polynesia in 2017 (1/3 singers) and 2018 (2/3 singers, both hybrid (singers 7 and 8)), as well as in Ecuador in 2018 (3/3 singers). Song type 2 (green; electronic supplementary material, figures S3 and S4) was described in French Polynesia in 2017 (2/3 singers) and 2018 (1/3 singers, part of hybrid singer 8). Song type 3 (orange; electronic supplementary material, figure S4) was sung by one singer (singer 9) in French Polynesia in 2018. Finally, song type 4 (grey; electronic supplementary material, figures S5 and S6) was identified in Ecuador in 2016 (3/3 singers) and 2017 (4/4 singers). Additionally, two singers in French Polynesia in 2018 (singers 7 and 8) sang hybrid songs combining themes from song types 1 (blue) and 3 (orange), and 1 (blue) and 2 (green), respectively (table 1; see electronic supplementary material, S1, for further details).

### 3.1. Song similarity in the central and eastern South Pacific

The LSI from hierarchically clustered and bootstrapped theme sequences for each singer revealed three major groupings (figure 2*b*, CCC = 0.962). Two initial branches were produced: one included song types 1 (blue), 2 (green) and 3 (orange) and the other song type 4 (grey). Song type 1 (blue) included all singers from French Polynesia 2016, two (of three) from 2017 and all Ecuador 2018 singers. Song types 2 (green; one singer from French Polynesia 2018) and 3 (orange; two singers from French Polynesia 2017) branched off the song type 1 (blue) cluster at a high level. Song type 4 (grey) included all Ecuador 2016 and 2017 singers and was entirely separated from the song type 1 (blue) cluster, which included the 2018 Ecuador singers. Both hybrid songs (singers 7 and 8) were grouped within song type 1 (blue; figure 2*b*). Singer 7 was placed on a separate branch to the rest of song type 1 (blue), while singer 8 was nested within the branch based on similarity in theme sequences (figure 2*b*).

**Figure 3.**
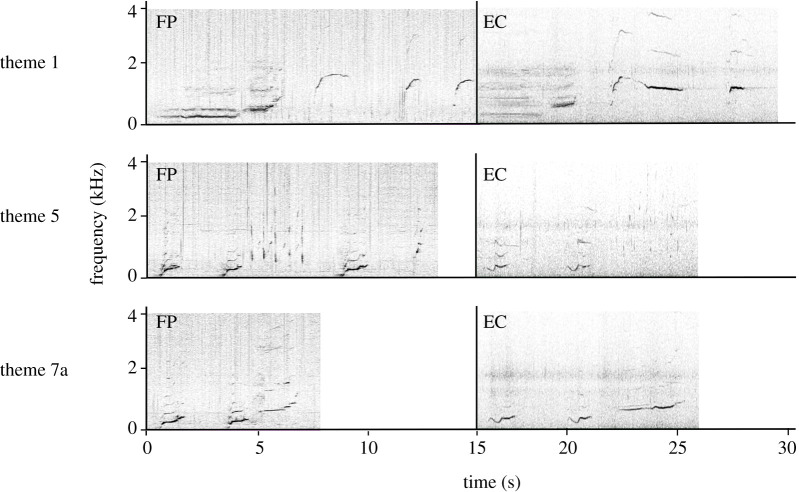
Spectrograms of matching themes 1, 5, 7a between French Polynesia (FP, left) and Ecuador (EC, right) from song type 1 (blue). Theme 1 consisted of the following units: long moan and ascending shriek, ascending whistle, short ascending whistle, ascending whistle (French Polynesia); long moan and ascending shriek, long n-shaped whistle, n-shaped whistle (Ecuador). Theme 5 consisted of the following units: two ascending moans, nine alternating high squeaks and squeaks, ascending moan, and ascending high squeak (French Polynesia); two modulated moans, and ten alternating high squeaks and squeaks (Ecuador). Theme 7a consisted of the following units: two ascending moans and an ascending shriek (French Polynesia); two modulated moans and an ascending shriek (Ecuador). See electronic supplementary material, table S1, for further information on matching theme sequences. Spectrograms were produced in Raven Pro 1.6 (fast Fourier transform 2048; Hann window, 50% overlap). The corresponding audio file is provided for each theme (electronic supplementary material, audio S1).

DSI based on the presence and sharing of themes regardless of their theme sequence produced a similar grouping to LSI (hierarchically clustered and bootstrapped; figure 2*c*, CCC = 0.966). Song types 1 (blue) and 4 (grey) clustered in a similar way to LSI, but with additional confidence in placement of singers within the dendrogram (based on AU and BP *p*-values). However, hybrid singer 7 (which combined themes from song types 1 and 3; blue/orange) was grouped with song type 3 (orange) singer 9, due to sharing theme 22 regardless of the sequence order of themes. Finally, regardless of the method and fine-scale placement of hybrid singers, all analyses indicated that there were four song types present across the central and eastern South Pacific over the course of the study (figure 3).

## 4. Discussion

Humpback whale song continues to spread east from French Polynesia in the central South Pacific to the Ecuadorian breeding ground in the eastern South Pacific. Three themes from song type 1 (blue) were documented in both French Polynesia in 2016, 2017 and 2018 as well as in Ecuador in 2018, confirming the hypothesis of delayed eastward transmission of song. Thus, the unidirectional song transmission in the South Pacific not only extends from Australia to French Polynesia [41,56], but a further 8000 km distance to Ecuador. This finding extends the geographical bounds of the horizontal cultural transmission of Southern Hemisphere humpback whale song and demonstrates vocal connectivity among populations that are 14 000 km apart (i.e. the distance from eastern Australia to Ecuador). It reiterates that the patterns of migration are written into the whales' song [58], and the potential for a full circumpolar transmission of song is highly plausible [12,70].

Recent agent-based cultural evolution models of global song transmission suggest the unidirectional song transmission is driven by differences in population sizes as songs spread from large to small populations [70]. We speculate that this may result in song transmission in different directions in other ocean basins dependent on population sizes (e.g. this allows the potential for songs to spread west across the South Indian Ocean from the large western Australian population). However, the global model also suggested that once a song revolution took hold, it continued to spread in a single direction [70]. We speculate this may allow a song to be passed in a stepwise fashion through all eleven Southern Hemisphere populations before returning to the origin population. The song would evolve during this time period (as is shown in the South Pacific [41]) and would be substantially different and thus considered a ‘new’ song by the time it transited the globe.

Song in both French Polynesia and Ecuador changed through progressive evolution and song revolutions, as is common for South Pacific breeding populations [41,56]. Progressive evolution occurs at a slower pace (over years) and changes are small, while song revolutions are identified when all themes of one song type are replaced by a novel one, and the origin of the novel song themes can be traced to a neighbouring breeding population [41,56]. Out of four song types identified in French Polynesia and Ecuador between 2016 and 2018, two song types resemble previously known song in western and central South Pacific breeding populations (electronic supplementary material, table S4). Song type 1 (blue) qualitatively resembled song themes previously recorded in French Polynesia, the Cook Islands and Tonga in 2015 (from [58]: figure 1 ‘light blue song’; electronic supplementary material, table S4). Five themes from the current study were visually and aurally similar to song themes identified in Owen *et al*. [58] (electronic supplementary material, table S4), with three themes present only in French Polynesia 2016–2018 (themes 3, 4 and 8), and two themes (themes 1 and 5) present in both French Polynesia 2016–2018 and Ecuador 2018 (table 1; electronic supplementary material, table S4). This suggests that humpback whales wintering in Ecuador are vocally connected with the western and central South Pacific breeding populations to at least Tonga. However, it is highly likely that the connection reaches back to the Australian breeding grounds based on previous studies that documented consistent song revolution events spreading from western to eastern Australia, onto New Caledonia, Tonga and finally to French Polynesia [40,41,57,58,64,67,68,72,82]. By contrast, the origin of song type 4 (grey) recorded in Ecuador in 2016 and 2017 preceding the song revolution in 2018 is unknown. The song themes did not resemble any of the themes described in French Polynesia or the wider South Pacific in previous studies. We hypothesize sporadic song sharing is occurring between the eastern and central South Pacific followed by periods of acoustic isolation and rapid evolution of the song making themes unrecognizable, and/or song sharing is occurring between the eastern South Pacific populations and Brazil (located on the east coast of South America) on a regular or sporadic basis.

We hypothesize that song type 1 (blue) spread from French Polynesia to Ecuador no earlier than 2016. The French Polynesian version of this song added three new themes between 2015 and 2016, and one of those themes (7a) was present in all song type 1 (blue) songs recorded in Ecuador in 2018 [58] (table 1; electronic supplementary material, table S1). Song may be transmitted between populations through three mechanisms [55]: song exchange on shared or partially shared feeding grounds or migratory routes, individuals using different breeding grounds in different years, or individuals moving between breeding grounds within a breeding season [55,57,58,60,64,72,83]. We hypothesize that the song was most likely transmitted between French Polynesia and Ecuador through song exchange on feeding grounds. Humpback whales from Ecuador (and its neighbouring population in breeding stock G, Colombia) feed near the Western Antarctic Peninsula (Antarctic Feeding Area I) based on genetic and photo-ID studies [84–86] (electronic supplementary material, figure S8). It is still uncertain, however, where the summer feeding grounds for French Polynesian whales are located, but there have been observations of some French Polynesian individuals and other South Pacific humpbacks near the Western Antarctic Peninsula [73,84,87]. Humpback whales sing mainly on breeding grounds and during migration, but multiple studies have recorded songs on foraging grounds around the globe [64,75,76,83]. This suggests that song learning is indeed possible during summer months when whales from different breeding populations may feed in similar areas (e.g. [64]). South Pacific breeding populations to the west of French Polynesia have been observed in Antarctic Feeding Areas V, VI and I [77,88], while overlap between foraging grounds has been found between Oceanian and Colombian breeding populations [84,89] (electronic supplementary material, figure S8). Genetic and photo-ID studies have observed individuals from French Polynesia and the Samoan islands near the West Antarctic Peninsula Area I [63,84,90], the typical feeding area for both the Colombian and Ecuadorian populations [84–86] (electronic supplementary material, figure S8). Clearly, there is potential for vocal exchange between French Polynesia and Ecuador due to overlap in the feeding grounds of at least a portion of the populations, and the occasional presence of song [83].

While song transmission on shared feeding grounds is the most likely scenario for song exchange in the current study, movement of whales from one breeding population to another between seasons has been observed between French Polynesia and other neighbouring populations in the central South Pacific [63] and, although rare, between French Polynesia and Colombia [91]. It cannot be ruled out that the song revolution from French Polynesia to Ecuador was the result of immigration of one or more whales into the Ecuadorian population. Finally, song may have spread east from French Polynesia through locations where whales have been sighted in small numbers (i.e. the Pitcairn Islands, Easter Island, Galapagos Islands [62]) in a stepping stone model. Future studies should incorporate opportunistic song recordings taken in remote central and eastern South Pacific locations to investigate this hypothesis. While song has continued to spread east, as predicted from previous empirical and modelling studies [41,70,92], we acknowledge that our sample size is small and spans only three years. However, these data produced a robust result in that song themes first present in French Polynesia appeared in Ecuador in a subsequent year replacing the existing song. Future studies should investigate whether this easterly transmission pattern holds through time given the apparent difference in population sizes between the smaller French Polynesian population and that of the larger stock G (Ecuador, Colombia, Costa Rica and Panama) [69], which may impact song dynamics [70]. Agent-based models indicate that songs are likely to spread from larger to smaller populations and as a result of rare interactions outside of the breeding grounds [70].

Finally, hybrid songs provide insights into the song learning process by which singers transition from singing an old song type to a new, novel song [60]. In the current study, two hybrid singers (singers 7 and 8) sung primarily song type 1 (blue) while mixing in themes from song types 2 (green) and 3 (orange) (table 1). Hybrid songs had only previously been documented a handful of times on the eastern Australian and French Polynesian breeding grounds and during the southbound migration past the Kermadec Islands in the western South Pacific [58,60]. However, while recording a hybrid song has historically been rare, season-long autonomous recorders in locations where song sharing, and learning, is occurring are more regularly capturing hybrid songs. Humpback whales are thought to learn songs through segmentation, and through the transition or substitution of themes influenced by sound sequence similarity [60]. Previously described hybrid songs contained themes of one song type that was commonly placed into the middle of the other song [60]. Here, singer 7 sang themes from song 1 (blue) and 3 (orange) early in the season, while singer 8 combined themes from song 1 (blue) and 2 (green) later in the season (table 1). This learning mechanism of picking up a novel song through segmentation has also been shown in songbirds near song dialect boundaries [93]. Hybrid songs occurred in village indigo bird (*Vidua chalybeate*) and orange-tufted sunbird (*Nectarinia osea*) males along dialect boundaries who displayed songs from either dialect [23,94]. While corn buntings also have distinct dialects and some males along dialect boundaries learn both, no song hybridization has been reported [12,24,25]. The dialects of corn buntings can be compared to humpback whale songs in a different aspect. Their dialects change from year to year with all males conforming to new versions much like how humpback whale songs progressively evolve [24,26]. In both species, changes to a current song through evolution are thought to occur through learning or production errors or innovation by individuals that are then adopted by the population [12,24,92]. By contrast, song revolutions in humpback whales are unlikely to be triggered by production errors [95] but rather represent the appearance of a new song type in a population [12]. A relevant comparison to song revolutions in songbirds may be that of white-throated sparrows (*Zonotrichia albicolis*) [96], where a novel song was documented spreading eastwards replacing the existing song across Canada [96]. This showed a replacement of song as documented for humpback whales in the South Pacific, but the time span over which song was replaced (two decades as opposed to merely two years in humpbacks) was different by an order of magnitude [41,96].

Here, we documented both song evolution and revolution, and the presence of hybrid singers which support previous studies on South Pacific humpback whale song dynamics [41,56,58,60,61,72]. The clear transmission of song themes from French Polynesia to Ecuador is the first evidence of song sharing between the two populations, although it is not known how this song will evolve in the next year, whether song revolutions in Ecuador occur repeatedly, and at what time intervals. The study of humpback whale song culture not only draws parallels to songbird song characteristics, but sheds light on the underlying mechanisms of social learning and cultural evolution in animals ranging from fish [97] to other cetacean species [10,98,99] through to humans [100]. Culture is an important aspect of animal communities that can increase the survival and reproductive success of individuals through socially learnt behaviours and traditions [6], and may increase their plasticity of responding to emerging threats.

## 5. Conclusion

Here, we documented the first evidence of eastward cultural transmission of songs from the central South Pacific breeding population of French Polynesia across to the eastern South Pacific breeding population of Ecuador. Three song themes were shared between the populations, with the song type being traced back as far as Tonga in the western South Pacific. This demonstrates that humpback whales are vocally connected across the ocean basin. Song transmission between French Polynesia and Ecuador is likely facilitated on overlapping feeding grounds around the West Antarctic Peninsula. This study extends our understanding of the extent of cultural transmission among humpback whale populations in the South Pacific and adds to unravelling the underlying mechanisms of song learning. Further studies are required to confirm whether song revolutions regularly spread from French Polynesia to Ecuador, and if so, at what interval. Further, to evaluate the extent of this cultural phenomenon, future studies should investigate whether songs continue to transmit eastwards from Ecuador (eastern South Pacific) to Brazil (western South Atlantic), and onwards around the Southern Hemisphere. Understanding this cultural phenomenon will provide valuable comparative perspectives to the evolution of complex communication including the evolution of human language and culture. As with humans, the patterns of migration are written into the songs of humpback whales.

## Data Availability

The dataset supporting this article (comprising raw song transcripts) has been uploaded as part of the electronic supplementary material (electronic supplementary material, S2) [101]. Song strings per singer are provided in table 1 and unit sequences for all phrase type set medians are provided in electronic supplementary material, table S1.
